# Pattern Visual Evoked Potential Changes in Diabetic Patients without Retinopathy

**DOI:** 10.1155/2017/8597629

**Published:** 2017-03-14

**Authors:** Ozgur Balta, Gulten Sungur, Mehmet Yakin, Nurten Unlu, Oyku Bezen Balta, Firdevs Ornek

**Affiliations:** ^1^Department of Ophthalmology, Dr. Nafiz Korez Sincan State Hospital, Ankara, Turkey; ^2^Department of Ophthalmology, Ankara Training and Research Hospital, Ankara, Turkey; ^3^Department of Family Medicine, Yildirim Beyazit University Faculty of Medicine, Ankara, Turkey

## Abstract

*Purpose.* To assess the different check sizes of pattern visual evoked potential (PVEP) in diabetic patients without retinopathy according to HbA1c levels and diabetes duration. *Methods.* Fifty-eight eligible patients with type 2 diabetes mellitus and 26 age- and sex-matched healthy controls were included in the study. Only the right eye of each patient was analyzed. All of the patients underwent a comprehensive ophthalmic examination, and the PVEPs were recorded. *Results.* There was a statistically significant difference in P100 latency in 1-degree check size and in N135 latency in 2-degree check size between controls and patient groups which have different HbA1c levels. There were statistically significant, positive, and weak correlations with diabetes duration and P100 latency in 7-minute and 15-minute check sizes and N135 latency in 15-minute check size. *Conclusions.* It was showed that there were prolongations in P100 latency only in 1-degree check size and in N135 only in 2-degree check size in diabetic patients without retinopathy. There was statistically significant correlation between diabetes duration and P100 and N135 latencies in different check sizes.

## 1. Introduction

In 2012, there were 1.5 million deaths worldwide directly caused by diabetes, and it was the eighth leading cause of death [1]. The World Health Organization (WHO) estimates that, globally, 422 million people aged over 18 years were living with diabetes in 2014. Good metabolic control significantly reduces the risk of development and progression of ocular and visual complications of both type 1 and type 2 diabetes [2]. Diabetic retinopathy (DR) was the cause of MSVI in 1.9% and of blindness in 2.6% globally in 2010 [3].

As found in many studies, controlling blood glucose significantly reduces the risk of visual complications of diabetes [2, 4–6]. In a study with a random sample of 914 diabetic patients, Kanh and Bradley found that there was a strong positive association between duration of diabetes and retinopathy [7]. Among diabetic patients with early onset, the retinopathy prevalence was 8% at the first 3 years, 25% at 5 years, 60% at 10 years, and 80% at 15 years. The prevalence of proliferative DR was 0% at the first 3 years and increased to 25% at 15 years among these patients [8].

Evoked potentials are noninvasive methods which can evaluate the electrophysiological response of the nervous system to different stimuli [9]. Visual evoked potentials (VEP) are used to examine the pathways through the optic nerves and brain. In the VEP method, visual fields are stimulated with a checkerboard visual stimulus, and the evoked response is recorded using surface recording electrodes. To not miss unilateral defects in the visual pathway, monocular stimulation is recommended in adults [10]. There are three stimulus protocols for recording VEP which are pattern VEP (PVEP), pattern onset/offset VEP, and flash VEP [11]. The PVEP is the preferential protocol because it has relatively low variability of waveform and peak latency both within a participant and study population [12]. There are three separate phases in the PVEP waveform: an initial negative deflection, a prominent positive deflection, and a later negative deflection. The peak latency and peak-to-peak amplitudes of these waves are measured [10].

The objectives of this study were to compare PVEP changes in diabetic patients without DR who had different HbA1c levels to healthy control participants without diabetes and to assess the correlation of PVEP responses with diabetes durations.

## 2. Methods

### 2.1. Participants and Ethical Considerations

This study was conducted in the Department of Ophthalmology at Ankara Training and Research Hospital, Turkey. The study was met with approval by the Institutional Review Board. Informed consents were obtained from all patients, and the Declaration of Helsinki was followed throughout the study.

Fifty-eight eligible patients with type 2 diabetes mellitus and 26 age- and sex-matched healthy controls were included in the study. The diagnosis of type 2 diabetes mellitus was based on the criteria of the World Health Organization (WHO). Exclusion criteria included BCVA worse than 20/20, high spherical or cylindrical > ± 1 dioptric refractive errors, pseudoexfoliation syndrome, diabetic retinopathy, history of uveitis, glaucoma, ocular trauma, previous intraocular surgery, and presence of systemic diseases, such as renal or hepatic dysfunction, obesity, and rheumatological diseases. Also, people who were currently smoking or using alcohol, and/or had prosthetic devices or electromagnetic field-generating devices, were excluded in the study.

Age, sex, duration of diabetes, and HbA1c levels were recorded. Diabetic patients were classified into two groups by HbA1c levels. Patients with 7% or less HbA1c levels were grouped as group I; patients with more than 7% were grouped as group II. Only the right eyes of each patient were included to the study.

All of the patients got a comprehensive ophthalmic examination, including medical history review, refraction, best-corrected visual acuity (BCVA), intraocular pressure (IOP) measured by the Goldmann applanation tonometer, and anterior and fundus segment examinations.

### 2.2. Pattern VEP

Retiscan Retipor 32 TM (Roland Consult, Wiesbaden, Germany) was used for recording according to the International Society for Clinical Electrophysiology of Vision (ISCEV) standards [12]. All tests were performed with the patients wearing the best refractive correction. PVEP was recorded monocularly for each patient. The active electrode was placed relative to bony landmarks, in proportion to the size of the head, according to the International 10/20 of electrode placement system with active electrode at Oz, reference electrode at Fz, and ground electrode at Fpz [13]. Pattern VEPs were recorded when the participants focused on the fixation point in the middle of the moving pattern of a checkerboard on the screen 1 meter away from them. Participants' fixations were closely followed by an experienced electrophysiology technician. Because of that which gives more information about the visual acuity of participants, PVEP was recorded using five different check sizes. The check sizes that were used were 120 (2 degrees), 60 (1 degree), 30, 15, and 7 minutes. Mean screen luminance was 100 cd/m^2^ with 99% contrast and a full-field display. The temporal frequency was 1.5 Hz (3 reversals per second). Mean luminance of the test room was 80 cd/m^2^, and recording conditions were kept according to the ISCEV standards. The amplifier band-pass filters were set at 1–50 Hz. To confirm the reproducibility of the waveform, two responses were recorded and superimposed.

### 2.3. Statistical Analysis

The Statistica version 10 (StatSoft Inc.) was used for statistical analyses. Descriptive statistics (median, minimum, maximum, and frequencies) were used to describe the baseline characteristics of the study groups. The Kruskal-Wallis test was used to compare nonnormally distributed quantitative variables between the study groups. Multiple comparisons of mean ranks for all groups were carried out as post hoc test, and Bonferroni adjustment was used for *p* values. To compare qualitative variables, the Pearson chi-square test was used. The association between diabetes duration and PVEP responses was analyzed by the Spearman correlation test in diabetic patients. *p* < 0.05 was accepted as statistically significant.

## 3. Results

This study included 58 patients with type 2 diabetes (36 males and 22 females) and 26 control subjects (16 males and 10 females). The mean age of the diabetes groups and control group was 49.4 ± 4.7, 51.9 ± 4.8, and 49.7 ± 7.6 years, respectively. Data on the age and gender of the subjects are presented in Table 1. There were no statistical differences in age and gender between control group and diabetic groups (*p* > 0.05).

According to glycemic statuses in diabetes, there were no statistically significant differences in N75 latency of PVEP between controls and patient groups in five different check sizes (Table 2); however, there were only statistically significant differences in 120-minute check between study group size in P100 latency (*p* = 0.042) (Table 3) and N135 latency (*p* = 0.013) (Table 4). In P100 latency, while it was found that there was no significant difference between controls and group I (*p* = 0.246), there was a statistically significant difference between controls and group II (*p* = 0.042). In N135 latency, whereas it was found that there was a significant difference between controls and group I (*p* = 0.018), there was no statistically significant difference between controls and group II (*p* = 0.058) (post hoc test).

It was found that there was no statistically significant correlation between diabetes duration and N75 latency, P100 latency, and N135 latency in 120-, 60-, and 30-minute check sizes. However, there was a statistically significant positive and weak correlation between diabetes duration and P100 latency in 15-minute check size (*R* = 0.322, *p* = 0.014).

**Figure 1:**
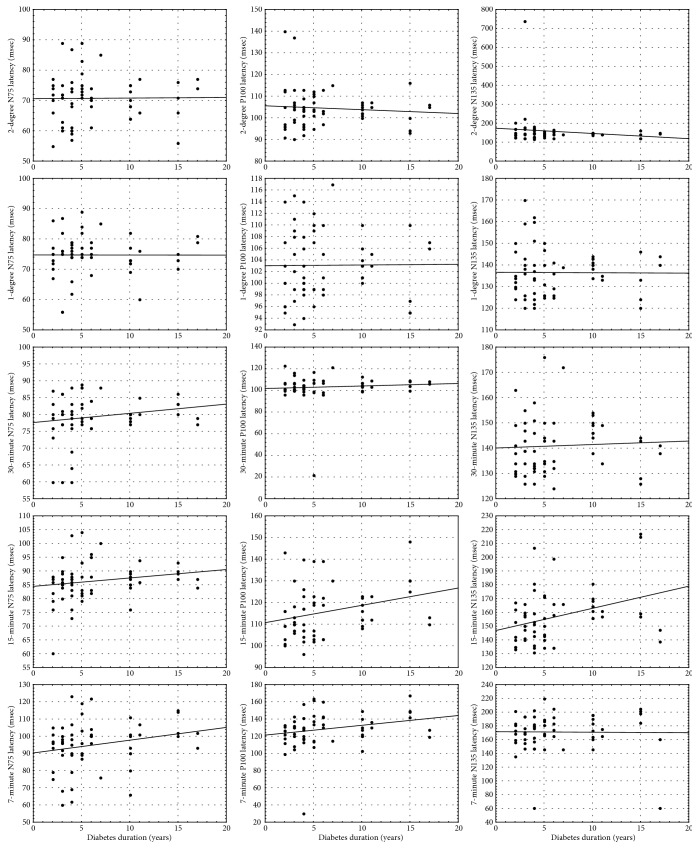
Correlation of N75, P100, and N135 latencies with diabetes duration.

Also, it was found that there was a statistically significant positive and weak correlation between diabetes duration and N135 latency in 15-minute check size (*R* = 0.330, *p* = 0.011). While there were no statistically significant correlations between diabetes duration and N75 latency and N135 latency, we found that there was a significant correlation between diabetes duration and P100 latency in 7-minute check size (*R* = 0.294, *p* = 0.025) (Table 5). These results were supported by correlation patterns of diabetes duration with P100 latency in 15-minute and 7-minute check sizes and with N135 latency in 15-minute check size in Figure 1.

## 4. Discussion

In 2010, DR was the fifth most common cause of preventable blindness and the fifth most common cause of moderate-severe visual impairment (MSVI) between 1990 and 2010. Also, the age-standardized prevalence of diabetic retinopathy causing MSVI had increased slightly from 1990 to 2010 [3]. In 2012, there were approximately 93 million people with diabetic retinopathy (DR), 17 million with proliferative DR, 21 million with diabetic macular edema, and 28 million with vision-threatening DR worldwide [14].

PVEP is a basic, sensitive, and practical method for assessing impulse conduction through the visual pathways. PVEP abnormalities have been described in DM, but increased P100 latency rate is ranging from 9% to 77% [15–17]. Abnormalities in PVEP latencies are much more important diagnostically than abnormalities in PVEP amplitude [10]. In this study, we assessed PVEP latencies rather than amplitudes.

It is hard to know that sex or sex-related risk factors may cause differences between males and females in diabetes and diabetic retinopathy. Nonetheless, sex must be considered in accurate viewpoints of diabetes and diabetic retinopathy in any case of etiology [18, 19]. The Wisconsin Epidemiologic Study of Diabetic Retinopathy reported that the severity of retinopathy was related to younger age at diagnosis, and the 10-year incidence of retinopathy and progression of retinopathy were the highest in the group disease onset before 30 years [20, 21]. In a study, Namperumalsamy et al. reported that older age (>50 years) was a significant risk factor for the prevalence of diabetic retinopathy in a South Indian community [22]. Also, sex has been recognized as an important variable which can affect the latency of PVEP parameters. In many studies, shorter P100 latencies in females were found [23]. On the other side, the stimulus parameters of PVEP (luminance, contrast, spatial, and temporal frequencies) modify the age-related changes in PVEP responses [24]. In our study, there was no statistical difference in sex and age between study groups. These findings were useful to interpret the results that sex and age were controlled as confounding factors between independent and dependent variables.

We found that there were prolongations in P100 latency only in 60-minute check size and in N135 only in 120-minute check size in diabetic patients without retinopathy. However, there was a statistically significant positive and weak correlation between diabetes duration and P100 latency in 15-minute and 7-minute check sizes and N135 latency in 15-minute check size.

Raman et al. studied with 25 diabetic patients and 15 age- and sex-matched controls to find whether the PVEP latencies are altered in diabetics or not and, if altered, whether it shows any correlation with the fasting blood sugar level. They did not report the check size of PVEP in their paper. They found that, while the P100 latencies in diabetic patients were significantly prolonged with a mean ± SD of 107.32 ± 4.14 in diabetics and 102.5 ± 3.77 in controls, N75 latency was 71.50 ± 5.3 in diabetics with a control of 70.4 ± 4.8 (the difference was not statistically significant). A positive correlation was documented between the delayed P100 latencies with the duration of disease and with the metabolic control of diabetes in their study [25].

Lana et al. found that the mean P100 latencies were significantly prolonged in 40 diabetic patients with a mean ± standard deviation of 109.87 ± 9.63 as compared with those in 50 controls (104.08 ± 3.31) (*p* = 0.014). They reported a positive correlation between fasting plasma glucose level and prolonged P100 latencies [26].

In a recent study conducted with 64 diabetic patients without retinopathy and 52 controls to detect the subclinical involvement of visual functions in diabetes by PVEP and to assess the value of the test in detecting preclinical form of diabetic retinopathy which could contribute greatly to the prevention of diabetic retinopathy complications, the significant prolongation of mean P100 latency was demonstrated in the diabetics as compared to that in the control group. Also, it was found that the duration of the illness alter the mean P100 latency [27].

In another study which included 40 diabetic patients including 20 subjects with nonproliferative diabetic retinopathy and 20 others without any retinopathy and compared to 40 age- and sex-matched normal nondiabetic controls, authors found that P100 wave latency was significantly longer in diabetic patients as compared to that in normal controls; both diabetic subjects without retinopathy and those with NPDR had significantly longer P100 latency than controls in 15-minute checkerboard pattern size. They reported that there was no statistically significant difference in N75 latency. No correlation was observed between N75 and P100 wave latencies and the duration of diabetes mellitus in their study [28].

However, Chopra et al. reported that PVEP P100 latency waves were prolonged in diabetic patients and also there was a significant correlation between the delay in the P100 latency and the duration of the disease in their study conducted on three groups (30 patients in each group) of type 2 DM (different durations of disease) and one group of 30 healthy age- and sex-matched controls [29].

## 5. Conclusion

We conducted the study to compare PVEP changes in diabetic patients without DR who had different HbA1c levels to healthy control participants without diabetes and to assess the correlation of PVEP responses with diabetes durations in five different check sizes. The check sizes used in the study generally were not reported in the study papers in PVEP in diabetes literature. We demonstrated that PVEP P100 waves were prolonged in 60-minute check size and in N135 only in 120-minute check size in diabetic patients without retinopathy. However, there was a statistically significant positive and weak correlation between diabetes duration and P100 latency in 15-minute and 7-minute check sizes and N135 latency in 15-minute check size.

## Figures and Tables

**Table 1:** Baseline characteristics of the study group.

	Controls (*n* = 26)	Group I (*n* = 26)	Group II (*n* = 32)	*p* value
*Gender*				
Male (%)	16 (61.5)	16 (61.5)	20 (62.5)	^∗^0.996
Female (%)	10 (38.5)	10 (38.5)	12 (37.5)	
*Age (years)*				
Mean ± SD	49.4 ± 4.7	51.9 ± 4.8	49.7 ± 7.6	†0.314
Median	50.0	50.0	51.5	
Minimum	42.0	42.0	40.0	
Maximum	60.0	59.0	60.0	

^∗^Chi-square test was used. †Kruskal-Wallis test was used.

**Table 2:** Median, minimum, and maximum N75 latency (msec) for 5 check sizes.

Check size	Controls (*n* = 26)			Group I (*n* = 26)			Group II (*n* = 32)			^∗^ *p* value
	Median	Min	Max	Median	Min	Max	Median	Min	Max	
120 min	70.0	16.0	77.0	70.5	55.0	87.0	72.0	55.0	87.0	0.469
60 min	75.0	69.0	81.0	74.0	60.0	89.0	75.0	56.0	86.0	0.582
30 min	79.0	74.0	86.0	80.0	69.0	88.0	79.5	60	89.0	0.698
15 min	84.5	66.0	86.0	87.0	76.0	95.0	86.0	60.0	104.0	0.723
7 min	99.0	83.0	119.0	97.5	62.0	122.0	95.5	60.0	123.0	0.346

^∗^Kruskal-Wallis test was used.

**Table 3:** Median, minimum, and maximum P100 latency (msec) for 5 check sizes.

Check size	Controls (*n* = 26)			Group I (*n* = 26)			Group II (*n* = 32)			^∗^ *p* value
	Median	Min	Max	Median	Min	Max	Median	Min	Max	
120 min	100.5	93.0	108.0	103.0	91.0	116.0	104.0	90.0	140.0	0.116
60 min	99.5	92.0	107.0	103.0	95.0	110.0	101.5	93.0	117.0	**0.042**
30 min	103.0	96.0	116.0	104.5	96.0	117.0	102.5	22.0	123.0	0.627
15 min	108.0	101.0	122.0	113.0	100.0	148.0	110.5	96.0	143.0	0.101
7 min	126.0	108.0	154.0	130.0	30.0	167.0	126.0	105.0	162.0	0.235

^∗^Kruskal-Wallis test was used.

**Table 4:** Median, minimum, and maximum N135 latency (msec) for 5 check sizes.

Check size	Controls (*n* = 26)			Group I (*n* = 26)			Group II (*n* = 32)			^∗^ *p* value
	Median	Min	Max	Median	Min	Max	Median	Min	Max	
120 min	133.5	117.0	166.0	145.0	120.0	176.0	142.0	116.0	737.0	**0.013**
60 min	135.0	120.0	150.0	137.0	120.0	170.0	134.5	120.0	162.0	0.413
30 min	134.0	123.0	160.0	143.0	124.0	154.0	136.5	126.0	176.0	0.267
15 min	146.0	130.0	176.0	158.5	134.0	217.0	150.0	131.0	207.0	0.097
7 min	172.0	95.0	190.0	170.5	61.0	220.0	175.0	60.0	203.0	0.830

^∗^Kruskal-Wallis test was used.

**Table 5:** The correlation of diabetes duration with N75, P100, and N135 latencies.

Check size	Diabetes duration with N75 latency		Diabetes duration with P100 latency		Diabetes duration with N135 latency	
	Correlation coefficient (R)	^∗^ *p* value	Correlation coefficient (R)	^∗^ *p* value	Correlation coefficient (R)	^∗^ *p* value
120 min	0.079	0.555	0.023	0.864	−0.154	0.250
60 min	−0.028	0.837	0.080	0.549	0.059	0.662
30 min	0.172	0.197	0.142	0.287	0.136	0.309
15 min	0.253	0.055	0.322	**0.014**	0.330	**0.011**
7 min	0.256	0.052	0.294	**0.025**	0.157	0.240

^∗^Spearman correlation test was used.
